# Elevated body roundness index increases the risk of cardiovascular disease in Chinese patients with circadian syndrome

**DOI:** 10.3389/fendo.2025.1532344

**Published:** 2025-02-25

**Authors:** Fenglin Zhang, Wenhua Shi, Jingwei Wen, Haiming Cao, Wenjing Xu, Taohua Lan, Wei Jiang, Xiankun Chen, Weihui Lu

**Affiliations:** ^1^ Second Clinical Medical College, Guangzhou University of Chinese Medicine, Guangzhou, Guangdong, China; ^2^ State Key Laboratory of Traditional Chinese Medicine Syndrome, Guangdong Provincial Hospital of Chinese Medicine, Guangzhou University of Chinese Medicine, Guangzhou, Guangdong, China; ^3^ Department of Cardiology, The Second Affiliated Hospital of Guangzhou University of Chinese Medicine, Guangzhou, Guangdong, China; ^4^ State Key Laboratory of Dampness Syndrome of Chinese Medicine, The Second Affiliated Hospital of Guangzhou University of Chinese Medicine, Guangzhou, Guangdong, China; ^5^ Academician Chen Keji Workstation, The Second Affiliated Hospital of Guangzhou University of Chinese Medicine, Guangzhou, Guangdong, China; ^6^ Key Unit of Methodology in Clinical Research, The Second Affiliated Hospital of Guangzhou University of Chinese Medicine, Guangzhou, Guangdong, China; ^7^ Chinese Medicine Guangdong Laboratory, Hengqin, Guangdong, China; ^8^ Guangdong Provincial Key Laboratory of Chinese Medicine for Prevention and Treatment of Refractory Chronic Diseases, Guangzhou, Guangdong, China

**Keywords:** cardiovascular disease, circadian syndrome, body roundness index, obesity, CHARLS

## Abstract

**Objective:**

The body roundness index (BRI) and circadian syndrome (CircS) are considered new risk factors for cardiovascular disease (CVD), yet it remains uncertain whether elevated BRI is associated with CVD incidence in CircS patients. In this study, we investigated the association between BRI and CVD occurrence among CircS participants.

**Methods:**

We conducted a retrospective cohort study involving 8,888 participants aged ≥45 years from the China Health and Retirement Longitudinal Study (CHARLS 2011-2020 wave). CircS was evaluated with a combination of the International Diabetes Federation (IDF) MetS, along with short sleep duration and depression. The threshold for CircS was established at ≥4. In the first phase, the receiver operating characteristic (ROC) curves were used to evaluate the accuracy of diagnosing CircS according to baseline BRI. During the 9-year follow-up, the associations between BRI and CVD incidence in CircS patients were explored by employing logistic regression, restricted cubic spline (RCS) analysis, and subgroup analysis.

**Results:**

BRI demonstrated an independent association with CircS, and multivariable-adjusted restricted cubic spline analyses suggested “J-shaped” associations between BRI and risk of CircS. BRI demonstrated better diagnostic performance in diagnosing CircS compared to general obesity indices such as ABSI (AUC: 0.617), BMI (AUC: 0.746), and WC (AUC: 0.722), with an AUC of 0.760. After a 9-year follow-up, BRI was found to be independently associated with the occurrence of CVD in CircS patients, and the associations between incident CVD and the second, third, and fourth BRI quartiles were 1.30 (95% CI: 0.99~1.69), 1.32 (95% CI: 1.01~1.72), and 1.59 (95% CI: 1.21~2.08), respectively, relative to the first BRI quartile. Then, we assessed the relationship between other obesity indices and the CVD occurrence, and likewise observed a significant effect in the fourth quartile.

**Conclusion:**

BRI was independently associated with CircS, outperforming obesity indices such as BMI and WC in identifying individuals with CircS. During the 9-year follow-up, elevated BRI levels was significantly associated with CVD incidence among CircS patients, especially in men. Thus, early identification of high-risk populations with CircS and elevated BRI levels may help promote healthy aging among middle-aged and elderly individuals.

## 1 Introduction

Cardiovascular disease (CVD) remains a major worldwide public health concern and leading contributor to non-communicable disease-related mortality, imposing a growing economic strain on both individuals and society (1, 2). In 2020, CVD surpassed cancer and other diseases to rank first in both incidence and mortality rates among residents of China (3). Early intervention in controlling risk factors is essential for preventing its occurrence.

The concept of circadian syndrome (CircS) has emerged to describe the relationship between circadian disruptions and various diseases, including obesity, dyslipidemia, hypertension, type 2 diabetes, sleep disorders, depression and non-alcoholic fatty liver disease (4). As living conditions have generally increased in China, the prevalence of CircS is now approaching 40%. As such, in recent years there has been an increasing focus on CircS due to its link with CVD (5). Thus, identifying specific risk factors for CVD among CircS individuals may contribute to reducing the future burden of CVD.

Adipose tissue regulates lipid storage and energy balance while serving as a vital modulator of metabolic health, facilitating bidirectional communication with other organs, including the cardiovascular system (6). Adipose tissue can be classified into either visceral fat tissue (VAT) or subcutaneous fat tissue (SAT) based on its anatomical location. Previous studies have demonstrated a significant association between visceral obesity and elevated cardiometabolic risk (7–9). However, whether elevated visceral adiposity in CircS patients is associated with increased risk of CVD remains poorly understood.

Magnetic resonance imaging (MRI) and computed tomography (CT) are widely regarded as the gold standards for assessing body fat and visceral fat tissue. However, their high costs prevent them from being used in routine clinical screening (10). The demand for a dependable and cost-effective measure of visceral adiposity has resulted in the creation of new indices that integrate anthropometric and biochemical evaluations: e.g., Thomas et al. introduced an innovative metric called the Body Roundness Index (BRI), which estimates body fat and visceral adipose tissue volume using waist circumference and height as inputs (11).

Recent research indicates a significant association between BRI and the risk of CVD (12–15). However, the relationship between BRI and CircS has yet to be explored, nor has the relationship between BRI and CVD in patients with CircS. To resolve this dearth in the literature, in the present study, we first investigate the relationship between BRI and CircS. Then, we evaluate the relationship between BRI and CVD among Chinese participants with CircS, focusing on middle-aged and elderly participants from the China Health and Retirement Longitudinal Study (CHARLS).

## 2 Materials and methods

### 2.1 Materials and methods

The China Health and Retirement Longitudinal Study (CHARLS), initiated in 2011, is a nationally representative cohort study that tracks individuals aged 45 and older from more than 20 provinces in China. Using a probability-proportional-to-size (PPS) sampling technique, the baseline survey involved over 17,000 participants from nearly 10,000 households in 450 villages and 150 counties. Participants were regularly followed up every two years through face-to-face interviews performed by the interviewer (using a computer to administer and record responses to survey questions). The follow-up survey waves were performed in 2013, 2015, 2018, and 2020. Conducted biennially by Peking University’s National School of Development, CHARLS adheres to strict ethical standards, including approval from Peking University’s Ethical Review Committee (IRB00001052-11015) and compliance with the Helsinki Declaration. The study provides critical insight into China’s aging population, with detailed methodological descriptions available in a prior publication (16).

**Figure 1:**
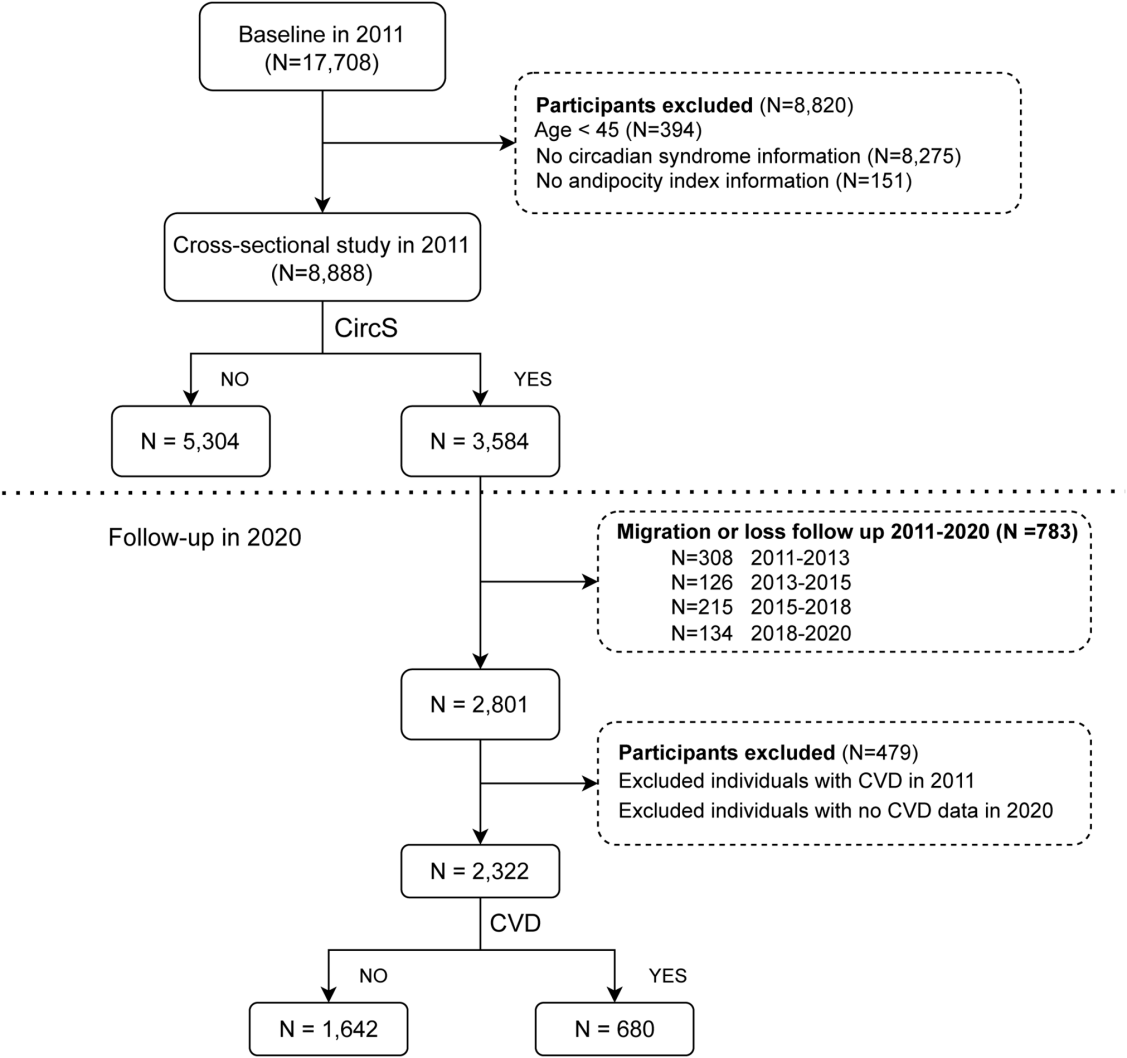
Flow diagram of study participants.

The present study was conducted based on data from five waves (2011, 2013, 2015, 2018 and 2020) in CHARLS. The inclusion criteria for this study were: 1) individuals aged 45 years or older in CHARLS 2011; and 2) who had data regarding circadian syndrome status. The exclusion criteria included: 1) absence of data about cardiovascular disease status at follow-up; 2) lack of age information; 3) absence of sex information; 4) unavailability of height, weight, waist circumference (WC) or adiposity index data. Ultimately, we included 8,888 individuals (3,584 of whom had been diagnosed with CircS) from the 2011 cross-sectional study to determine the relationship between BRI and CircS. After 9 years of follow-up, 2,322 CircS participants were included in a longitudinal cohort study using data from 2011 to 2020 to further explore the relationship between BRI and CVD incidence, while 783 participants moved or were otherwise lost to follow up, and 479 participants were excluded because of CVD at baseline or missing data through 2020. **Figure 1** illustrates the comprehensive selection procedure.

### 2.2 Data collection

At baseline, trained interviewers collected data on characteristics by employing a predefined questionnaire. Socio-demographic variables included age, sex, education, marital status, and residence. Health-related factors included body mass index (BMI), smoking and drinking status (current, former or never), self-reported physician-diagnosed dyslipidemia, hypertension, diabetes or high blood glucose, cardiovascular disease and chronic kidney disease.

Physical examination data were also recorded, including WC, weight, height, systolic blood pressure (SBP), and diastolic blood pressure (DBP). Additionally, biochemical indicators were documented, involving fasting blood glucose, glycosylated hemoglobin, total cholesterol, triglycerides, high-density lipoprotein cholesterol, low-density lipoprotein cholesterol, and hypersensitive C-reactive protein levels. Participants were classified as “physically active” if they engaged in moderate activity for at least 30 minutes five times a week or vigorous activity for at least 20 minutes three times a week; the others were classified as “physically inactive” (17). Participants were given the instructions to fast 8–12 hours before blood sampling. Bioassays were conducted at national or local Centers for Disease Control that complied with standardized quality control standards, while blood samples were preserved at −70°C. Finally, night-time sleep duration and daytime napping were measured, and the Center for Epidemiologic Studies-Depression scale (CES-D) score was determined.

### 2.3 Assessing circadian syndrome status

The CircS criteria, established and verified in CHARLS, encompassed abdominal obesity (WC ≥ 85 cm in men, and ≥ 80 cm in women), hypertension (SBP ≥ 130 mmHg and/or DBP ≥ 85 mmHg, or drug treatment for hypertension), hyperglycemia (≥100 mg/dl or drug treatment for elevated glucose), high triglycerides (≥150 mg/dl or drug treatment for high triglycerides), low HDL cholesterol (<40 mg/dl or drug treatment for low HDL cholesterol), depression and short sleep duration (<6 h/day) (5, 18, 19). CircS was diagnosed in participants who satisfied ≥ 4 of these criteria.

WC was obtained at the umbilicus level with participants in a standing position. Blood pressure was measured three times, using the SBP and DBP averages (5). Symptoms of depression were evaluated using the 10-item CES-D scale, with scores ≥10 indicating depressive symptoms (20). Total sleep duration, calculated by summing nap and nighttime sleep durations from a questionnaire, was considered short if it was less than 6 hours per day (19). CircS was assessed based on seven components—the 5 components used to define MetS as well as short sleep duration and depression (5, 19, 21).

### 2.4 Adiposity index calculation

Any values for WC, weight and height that were less than the 1% percentile cut-off were replaced with 1% percentile values to avoid the influence of outliers. Lacking uniform classification criteria, we divided the BRI into quartiles. We used the following formulas to measure the adiposity indices.


$$BMI=\frac{Weight}{Height^{2}}$$

$$ABSI=\frac{WC}{Height^{\frac{1}{2}}\times BMI^{\frac{2}{3}}}$$

$$CI=\frac{WC(m)}{0.019\sqrt{\frac{weight(kg)}{height\left(m\right)}}}$$

$$\text{BRI}=364.2-365.5\sqrt{1-\left(\frac{WC÷{\left(2\pi \right)}^{2}}{{\left(0.5\times Height\right)}^{2}}\right)}$$

$$\begin{array}{c} \text{CAVI}=-267.93\;+\;0.68\times \;age\;+0.03\times BMI+4\times WC+22\times logTG\\ \left(mmol/L\right)-16.32\times HDL_{\left(mmol/L\right)}(men) \end{array}$$

$$\begin{array}{c} \text{CAVI}=-187.32\;+\;1.71\times \;age\;+4.23\times BMI+1.12\times WC+39.76\times logTG\\ \left(mmol/L\right)-11.66\times HDL_{\left(mmol/L\right)}(women) \end{array}$$

### 2.5 CVD event assessment

The CVD included heart disease and stroke. Similar to previous studies (18, 22), newly diagnosed CVD was assessed through responses to the following questionnaire item: “Have you been diagnosed with heart attack, coronary heart disease, angina, congestive heart failure, other heart problems, or stroke by a doctor?”

### 2.6 Statistical analysis

All normally distributed continuous variables were reported as mean ± SD, and skewed continuous variables were presented as median and interquartile range. Categorical variables were presented as frequencies (%). Participants without CircS were compared to those with CircS based on the baseline characteristics. In addition, we categorized patients into quartiles according the baseline BRI levels in order to illustrate CircS patients’ baseline characteristics. We used Chi-square or Fisher’s exact (categorical variables), a one-way ANOVA test (normal distribution), or a Kruskal-Whallis H-test (skewed distribution) to test for differences among different groups.

We employed multivariate logistic regression models to evaluate the relationship between BRI and CircS diagnosis utilizing several multivariable models with various levels of adjustment. Three models were estimated: Model 1 was unadjusted; Model 2 adjusted for age and sex; Model 3 included the same adjustments as Model 2 with further adjustments for education level, residence, marital status, smoking, drinking, physical activity, blood pressure, HbA1c, history of hypertension, dyslipidemia, diabetes, cardiovascular disease and chronic kidney disease. We selected these confounders on the basis of previous research (12, 13), significant covariates in the univariate analysis, or their associations with the outcomes of interest or a change in effect estimate exceeding 10%. All variables included in the models met the criteria of tolerance (> 0.1) and had a variance inflation factor < 10. We assessed the diagnostic performance of BRI in determining CircS in comparison to adiposity indices employing ROC curve analyses, and utilized a restricted cubic spline to assess the dose-response relationship between BRI and CircS.

We employed multivariate logistic regression models to assess the relationship between CVD incidence among CircS patients and BRI, along with other adiposity indicators, after a 9-year follow-up. We estimated three models: Model 1 was unadjusted; Model 2 adjusted for age and sex; Model 3 included the same adjustments as Model 2 with further adjustments for education level, residence, marital status, smoking, drinking and physical activity. We selected these confounders on the basis of previous research (5, 18), significant covariates in the univariate analysis, or their associations with the outcomes of interest or a change in effect estimate exceeding 10%. All variables included in the models met the criteria of tolerance > 0.1 and variance inflation factor < 10. We conducted stratified analyses by sex and age, producing *P* values for interactions, and sensitivity analysis to assess the results’ robustness by excluding any participants who experienced CVD during the first 2 years. All analyses were conducted with R Statistical Software (Version 4.2.2, http://www.R-project.org, The R Foundation) and the Free Statistics analysis platform (Version 1.9). A two-sided *P* value < 0.05 was considered statistically significant.

## 3 Results

### 3.1 Study participants’ characteristics

**Table 1** presents the characteristics of the participants according to CircS. After satisfying the above inclusion criteria, we analyzed a total of 8,888 participants at the baseline (**Figure 1**).

**Table 1:** Participants’ baseline characteristics.

	Total (n = 8,888)	Non-CircS (n = 5,304)	CircS (n = 3,584)	*P* value
Age, y	59.4 ± 9.3	58.9 ± 9.4	60.0 ± 9.1	< 0.001
Sex, n (%)				< 0.001
Male	4,185 (47.1)	2,900 (54.7)	1,285 (35.9)	
Female	4,703 (52.9)	2,404 (45.3)	2,299 (64.1)	
Marital status, n (%)				0.464
Married	7,875 (88.6)	4,764 (89.8)	3,111 (86.8)	
Single, divorced or widowed	1,013 (11.4)	540 (10.2)	473 (13.2)	
Education, n (%)				0.005
Primary school and below	6,176 (69.5)	3,624 (68.3)	2,552 (71.2)	
Junior high school	1,809 (20.4)	1,104 (20.8)	705 (19.7)	
Senior high school and above	903 (10.2)	576 (10.9)	327 (9.1)	
Residence, n (%)				0.291
Rural	7,305 (82.2)	4,484 (84.6)	2,821 (78.7)	
Urban	1,581 (17.8)	818 (15.4)	763 (21.3)	
Smoking, n (%)				< 0.001
Smoker	2,734 (30.8)	1,922 (36.2)	812 (22.7)	
Former smoker	816 (9.2)	479 (9)	337 (9.4)	
Never smoked	5,337 (60.1)	2,902 (54.7)	2,435 (67.9)	
Alcohol consumption, n (%)				< 0.001
Alcohol drinker	2,925 (32.9)	2,000 (37.7)	925 (25.8)	
Former drinker	746 (8.4)	420 (7.9)	326 (9.1)	
Never drank alcohol	5,217 (58.7)	2,884 (54.4)	2,333 (65.1)	
Physical activity, n (%)				0.004
Inactive	6,734 (75.8)	3,905 (73.6)	2,829 (78.9)	
Active	2,154 (24.2)	1,399 (26.4)	755 (21.1)	
SBP, mmHg	130.7 ± 21.5	125.9 ± 20.2	137.8 ± 21.6	< 0.001
DBP, mmHg	75.9 ± 12.2	73.5 ± 11.6	79.4 ± 12.1	< 0.001
HbA1c, %	5.3 ± 0.8	5.2 ± 0.7	5.4 ± 1.0	< 0.001
LDL-C, mg/dL	116.8 ± 35.1	116.0 ± 32.2	118.0 ± 39.0	0.007
TC, mg/dL	194.0 ± 39.0	189.6 ± 35.7	200.4 ± 42.5	< 0.001
TG, mg/dL	106.2 (75.2, 154.9)	87.6 (66.4, 115.9)	157.5 (106.2, 217.7)	< 0.001
CRP, mg/L	1.1 (0.6, 2.2)	0.9 (0.5, 1.8)	1.3 (0.7, 2.7)	< 0.001
Chronic disease, n (%)				
Hypertension	2,237 (25.3)	779 (14.7)	1,458 (40.9)	< 0.001
Dyslipidemia	828 (9.5)	209 (4)	619 (17.6)	< 0.001
Diabetes	517 (5.9)	144 (2.7)	373 (10.5)	< 0.001
Cardiovascular disease	1,065 (12.0)	466 (8.8)	599 (16.7)	< 0.001
Chronic kidney disease	591 (6.7)	346 (6.5)	245 (6.8)	0.582
Adiposity indicators, n (%)				
BMI	23.6 ± 3.9	22.5 ± 3.5	25.2 ± 3.9	< 0.001
WC	84.4 ± 12.5	80.9 ± 12.0	89.7 ± 11.3	< 0.001
CI	1.3 ± 0.1	1.2 ± 0.2	1.3 ± 0.1	< 0.001
ASBI	0.1 ± 0.0	0.1 ± 0.0	0.1 ± 0.0	< 0.001
BRI	4.2 ± 1.5	3.7 ± 1.4	4.9 ± 1.4	< 0.001

CircS, circadian syndrome; SBP, systolic blood pressure; DBP, diastolic blood pressure; HbA1C, glycosylated hemoglobin A1c; LDL-C, low-density lipoprotein cholesterol; TC, total cholesterol; TG, triglycerides; CRP, C-reactive protein; BMI, body mass index; WC, waist circumference; CI, conicity index; ABSI, A body shape index; BRI, body-roundness index.

The mean and standard deviation for age in the study population was 59.4 ± 9.3 years, and 3,584 (40.3%) participants had been diagnosed with CircS at baseline. Individuals with CircS were more likely to be older, female, have lower literacy, be physically inactive, and have higher blood pressure, TC, TG, LDL-C, HbA1c, CRP and adiposity index scores (all *P <*0.05, **Table 1**).

### 3.2 The associations between BRI and CircS at the baseline

As the BRI increased, there was a corresponding increase in the prevalence of CircS. **Table 2** demonstrates the correlation between BRI and CircS. In the unadjusted model, for each additional SD increase in BRI, the risk of CircS increased by 1.76. In all of the adjusted models, BRI was independently associated with CircS, and the adjusted odds ratios were 2.31 (2.17~2.45), and 2.14 (2~2.28), respectively.

**Table 2:** Associations between baseline BRI and CircS.

	Case, %	OR (95% CI)					
		Model 1	*P* value	Model 2	*P* value	Model 3	*P* value
Per SD increase	3,584 (40.3)	2.76 (2.61~2.92)	<0.001	2.31 (2.17~2.45)	<0.001	2.14 (2.01~2.28)	<0.001
Quartiles							
Q1 (< 3.19)	275 (12.4)	Reference		Reference		Reference	
Q2 (3.19 ~ 4.04)	643 (29)	2.89 (2.47~3.38)	<0.001	2.67 (2.27~3.14)	<0.001	2.72 (2.30~3.23)	<0.001
Q3 (4.04 ~ 5.08)	1,159 (52)	7.69 (6.61~8.94)	<0.001	6.28 (5.36~7.34)	<0.001	5.91 (5.01~6.97)	<0.001
Q4 (> 5.08)	1,507 (67.9)	14.97 (12.83~17.47)	<0.001	10.21 (8.68~12.02)	<0.001	8.68 (7.31~10.32)	<0.001
*P* for trend			<0.001		<0.001		<0.001

BRI, body-roundness index; CircS, circadian syndrome; OR, odds ratio; CI, confidence interval.

Model 1: unadjusted. Model 2: adjusted for age, sex, education level, residence, marital status, smoking status, alcohol consumption, physical activity and blood pressure. Model 3: adjusted for age, sex, education level, residence, marital status, smoking status, alcohol consumption, physical activity, blood pressure, HbA1c, history of hypertension, dyslipidemia, diabetes, cardiovascular disease and chronic kidney disease.

When assessed as quartiles, there was a significant association between BRI and CircS in the second, third, and fourth quartiles—even after adjusting for all confounding factors (adjusted OR: 2.72, 95% CI: 2.3~3.23; adjusted OR: 5.91, 95% CI: 5.01~6.97; adjusted OR: 8.68, 95% CI: 7.31~10.32, respectively). Additionally, multivariable-adjusted restricted cubic spline analyses suggested “J-shaped” associations between BRI and risk of CircS (**Supplementary Figure 1**; *P* for nonlinearity < 0.05).

We used ROC curve analysis to compare the diagnostic efficacy of BRI with other adiposity indices in detecting CircS (**Supplementary Figure 2**). In diagnosing CircS, the BRI demonstrated the highest AUC values (AUC: 0.760, 95% CI: 0.750 ~ 0.770), exceeding those of ABSI (AUC: 0.617, 95% CI: 0.606 ~ 0.629), CI (AUC: 0.649, 95% CI: 0.683 ~ 0.705), BMI (AUC: 0.722, 95% CI: 0.711 ~ 0.733) and WC (AUC: 0.746, 95% CI: 0.736 ~ 0.756). **Table 3** presents the diagnostic performance of each anthropometric index in identifying CircS, encompassing sensitivity, specificity, and corresponding optimal cut-off values. BRI exhibited the highest Youden indices (0.415) for identifying CircS, with an optimal cut-off of 3.96.

**Table 3:** Cut-off between area under the curve, sensitivity, and specificity for adiposity indices to detect circadian syndrome.

	BMI	WC	ABSI	CI	BRI
AUC	0.722	0.746	0.617	0.694	0.76
95% CI	0.711 ~ 0.733	0.736 ~ 0.756	0.606 ~ 0.629	0.683 ~ 0.705	0.750 ~ 0.770
*p* value	<0.001	<0.001	<0.001	<0.001	<0.001
Cut-off point	23.20	84.95	0.082	1.277	3.961
Youden Index	0.351	0.409	0.184	0.310	0.415
Sensitivity	0.708	0.741	0.688	0.719	0.769
Specificity	0.643	0.668	0.496	0.592	0.646

AUC, area under the curve; CircS, circadian syndrome; BMI, body mass index; WC, waist circumference; CI, conicity index; ABSI, A body shape index; BRI, body-roundness index.

### 3.3 CircS patients’ baseline characteristics divided into BRI quartiles

**Table 4** presents the characteristics of the participants with CircS according to the BRI quartiles (Q1: < 4.00, Q2: 4.00 ~ 4.82, Q3: 4.82 ~ 5.65, and Q4: > 5.65). Among these 2,322 participants with CircS, the prevalence of heart disease, stroke, and CVD was 10.3% (239/2,322), 22.4% (520/2,322) and 29.3% (680/2,322), respectively. Individuals with higher BRI were more likely to be older, female, have lower literacy, higher BMI, blood pressure, TC, LDL-C, and HbA1c, and have a higher likelihood of being diagnosed with hypertension (all *P <*0.05, **Table 4**).

**Table 4:** CircS patients’ baseline characteristics divided into BRI quartiles.

	Total (n = 2,322)	Q1 (n = 580)	Q2 (n = 580)	Q3 (n = 581)	Q4 (n = 581)	*P* value
Age, y	58.4 ± 8.3	58.1 ± 8.0	57.3 ± 8.0	58.4 ± 8.4	59.9 ± 8.6	< 0.001
Sex, n (%)						< 0.001
Male	797 (34.3)	294 (50.7)	227 (39.1)	180 (31)	96 (16.5)	
Female	1,525 (65.7)	286 (49.3)	353 (60.9)	401 (69)	485 (83.5)	
Marriage, n (%)						0.464
Married	2,094 (90.2)	526 (90.7)	531 (91.6)	518 (89.2)	519 (89.3)	
Single, divorced or widowed	228 (9.8)	54 (9.3)	49 (8.4)	63 (10.8)	62 (10.7)	
Education, n (%)						0.005
Primary school and below	1,647 (70.9)	399 (68.8)	396 (68.3)	410 (70.6)	442 (76.1)	
Junior high school	472 (20.3)	125 (21.6)	116 (20)	124 (21.3)	107 (18.4)	
Senior high school and above	203 (8.7)	56 (9.7)	68 (11.7)	47 (8.1)	32 (5.5)	
Residence, n (%)						0.291
Rural	1,930 (83.1)	478 (82.4)	477 (82.2)	477 (82.1)	498 (85.7)	
Urban	392 (16.9)	102 (17.6)	103 (17.8)	104 (17.9)	83 (14.3)	
Smoking, n (%)						< 0.001
Current smoker	519 (22.4)	198 (34.1)	144 (24.8)	118 (20.3)	59 (10.2)	
Former smoker	172 (7.4)	51 (8.8)	46 (7.9)	43 (7.4)	32 (5.5)	
Never smoked	1,631 (70.2)	331 (57.1)	390 (67.2)	420 (72.3)	490 (84.3)	
Alcohol consumption, n (%)						< 0.001
Alcohol drinker	636 (27.4)	222 (38.3)	173 (29.8)	147 (25.3)	94 (16.2)	
Former drinker	182 (7.8)	44 (7.6)	57 (9.8)	43 (7.4)	38 (6.5)	
Never drank alcohol	1,504 (64.8)	314 (54.1)	350 (60.3)	391 (67.3)	449 (77.3)	
BMI category, n (%)						< 0.001
Underweight	51 (2.2)	41 (7.1)	4 (0.7)	4 (0.7)	2 (0.3)	
Normal weight	839 (36.3)	423 (73.4)	253 (43.8)	123 (21.2)	40 (6.9)	
Overweight or obese	1,422 (61.5)	112 (19.4)	321 (55.5)	453 (78.1)	536 (92.7)	
Physical activity, n (%)						0.004
Inactive	1,772 (76.3)	418 (72.1)	443 (76.4)	440 (75.7)	471 (81.1)	
Active	550 (23.7)	162 (27.9)	137 (23.6)	141 (24.3)	110 (18.9)	
SBP, mmHg	136.6 ± 21.0	133.5 ± 20.6	134.6 ± 20.7	137.2 ± 20.7	141.2 ± 21.2	< 0.001
DBP, mmHg	79.4 ± 12.0	77.9 ± 12.3	78.7 ± 11.7	79.9 ± 12.0	81.0 ± 11.8	< 0.001
HbA1c, %	5.4 ± 0.9	5.2 ± 0.8	5.3 ± 0.8	5.5 ± 1.0	5.5 ± 1.0	< 0.001
LDL-C, mg/dL	117.0 ± 38.7	111.8 ± 37.0	116.3 ± 38.3	118.1 ± 39.5	121.7 ± 39.2	< 0.001
TC, mg/dL	200.2 ± 42.1	195.4 ± 39.8	198.9 ± 40.7	201.6 ± 45.6	204.9 ± 41.6	0.001
TG, mg/dL	160.2 (108.0, 222.1)	161.5 (103.5, 220.6)	158.4 (106.0, 207.5)	161.1 (111.5, 228.3)	161.1 (109.7, 223.9)	0.391
CRP, mg/L	1.3 (0.7, 2.5)	1.0 (0.6, 2.2)	1.1 (0.6, 2.1)	1.4 (0.8, 2.5)	1.6 (1.0, 3.4)	< 0.001
Chronic disease, n (%)						
Hypertension	785 (34.0)	152 (26.3)	155 (27.1)	204 (35.2)	274 (47.2)	< 0.001
Chronic kidney disease	127 (5.5)	43 (7.4)	36 (6.2)	29 (5)	19 (3.3)	0.014

CircS, circadian syndrome; BRI, body roundness index; BMI, body mass index; SBP, systolic blood pressure; DBP, diastolic blood pressure; HbA1C, glycosylated hemoglobin A1c; LDL-C, low-density lipoprotein cholesterol; TC, total cholesterol; TG, triglycerides; CRP, C-reactive protein.

### 3.4 The longitudinal association between BRI and CVD in CircS patients, 2011-2020

The associations between BRI and different CVD components are shown in **Table 5**. In all adjusted models, for each additional SD increase, BRI was independently associated with CVD occurrence, and the adjusted odds ratios were 1.16 (1.06~1.28), and 1.14 (1.04~1.26), respectively. When assessed as quartiles, the associations between incident CVD and the second, third, and fourth BRI quartiles were 1.30 (95% CI: 0.99~1.69), 1.32 (95% CI: 1.01~1.72), and 1.59 (95% CI: 1.21~2.08), respectively, relative to the first BRI quartile. However, only the fourth BRI quartile was significantly associated with the incidence of heart disease and stroke, with ORs of 1.09 (95% CI: 0.98~1.21) and 1.18 (95% CI: 1.03~1.36). To evaluate whether there was a dose-response relationship between BRI and CVD incidence in CircS patients, we used a smoothing function analysis. Adjusted smoothed plots suggested a straightforward linear relationship between BRI and CVD incidence (**Supplementary Figure 3**, *P* for non-linearity = 0.635). As the BRI level increases, the risk of CVD shows an upward trend.

**Table 5:** CVD incidence according to CircS patients’ baselines BRI, 2011–2020.

Outcome	Case, %	OR (95% CI)					
		Model 1	*P* value	Model 2	*P* value	Model 3	*P* value
CVD							
Per SD increase	680 (29.3)	1.19 (1.09~1.31)	<0.001	1.16 (1.06~1.28)	0.002	1.14 (1.04~1.26)	0.005
Quartiles							
Q1 (< 4.00)	137 (23.6)	Reference		Reference		Reference	
Q2 (4.00 ~ 4.82)	167 (28.8)	1.31 (1.01~1.70)	0.045	1.32 (1.01~1.72)	0.039	1.30 (0.99~1.69)	0.056
Q3 (4.82 ~ 5.65)	172 (29.6)	1.36 (1.05~1.77)	0.021	1.34 (1.03~1.74)	0.032	1.32 (1.01~1.72)	0.043
Q4 (> 5.65)	204 (35.1)	1.75 (1.35~2.26)	<0.001	1.66 (1.27~2.17)	<0.001	1.59 (1.21~2.08)	0.001
*P* for trend			<0.001		<0.001		0.001
Heart disease							
Per SD increase	520 (22.4)	1.15 (1.05~1.27)	0.005	1.10 (0.99~1.21)	0.074	1.08 (0.98~1.20)	0.128
Quartiles							
Q1 (< 4.00)	107 (18.4)	Reference		Reference		Reference	
Q2 (4.00 ~ 4.82)	128 (22.1)	1.25 (0.94~1.67)	0.125	1.23 (0.92~1.64)	0.166	1.21 (0.91~1.62)	0.193
Q3 (4.82 ~ 5.65)	129 (22.2)	1.26 (0.95~1.68)	0.112	1.19 (0.89~1.59)	0.248	1.17 (0.87~1.57)	0.290
Q4 (> 5.65)	156 (26.9)	1.62 (1.23~2.14)	0.001	1.44 (1.08~1.92)	0.014	1.39 (1.04~1.86)	0.028
*P* for trend			0.001		0.024		0.045
Stroke							
Per SD increase	239 (10.3)	1.15 (1.01~1.32)	0.036	1.18 (1.03~1.36)	0.020	1.17 (1.02~1.35)	0.026
Quartiles							
Q1 (< 4.00)	48 (8.3)	Reference		Reference		Reference	
Q2 (4.00 ~ 4.82)	57 (9.8)	1.21 (0.81~1.81)	0.358	1.29 (0.86~1.93)	0.220	1.25 (0.83~1.88)	0.283
Q3 (4.82 ~ 5.65)	62 (10.7)	1.32 (0.89~1.97)	0.165	1.42 (0.95~2.13)	0.084	1.42 (0.95~2.12)	0.091
Q4 (> 5.65)	72 (12.4)	1.57 (1.07~2.30)	0.022	1.74 (1.16~2.60)	0.007	1.69 (1.12~2.53)	0.012
*P* for trend			0.020		0.007		0.010

CVD, cardiovascular disease; CircS, circadian syndrome; BRI, body-roundness index; OR, odds ratio; CI, confidence interval.

Model 1: unadjusted; Model 2: adjusted for age, sex; Model 3: adjusted for age, sex, education level, residence, marital status, smoking status, alcohol consumption, physical activity.

We also explore the relationship between other obesity indices and CVD occurrence (**Supplementary Table 1**). After adjusting for confounding factors, compared with the first quantile, the fourth quartiles of BMI, WC, and CVAI were significantly associated with the occurrence of CVD (OR: 1.54, 95% CI: 1.18~2; OR: 1.72, 95% CI: 1.33~2.22; OR: 1.34, 95% CI: 1.03~1.73, respectively). To evaluate whether there was a dose-response relationship between obesity indices and CVD incidence in CircS patients, we used a smoothing function analysis. After adjusting for potential confounding factors, we observed a linear relationship between BMI, CVAI and CVD (**Supplementary Figures 3, 4**; *P* for non-linearity >0.05). Additionally, the risk of CVD incidence was linearly associated with BMI and CVAI (**Supplementary Figures 4, 5**), and followed a J -shaped curve with respect to WC (**Supplementary Figure 6**).

### 3.5 Subgroup analysis

**Figure 2:**
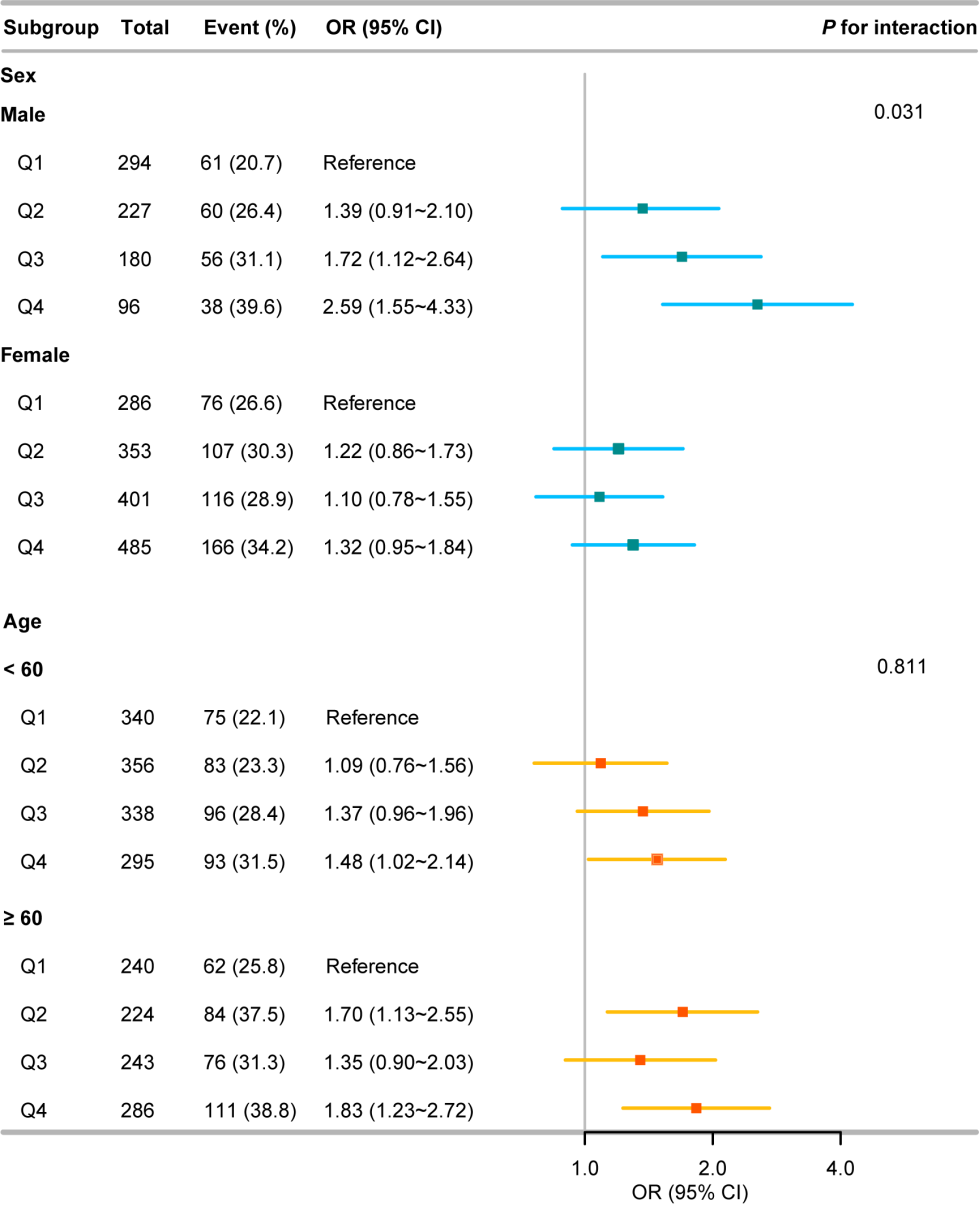
Forest diagram of OR after adjusting for confounding factors of sex and age.

**Table 6** shows results stratified by sex, and age group. We found similar associations between BRI and CVD in most of the subgroup analyses. In subgroup analysis, the third and fourth BRI quartiles were significantly associated with higher CVD incidence in men (adjusted OR: 1.72, 95% CI: 1.12~2.64; adjusted OR: 2.59, 95% CI: 1.55~4.33, respectively). However, we found similar positive associations among women, with no significant differences. Furthermore, the significant associations between the fourth BRI quartiles and CVD incidence in the middle-aged and elderly adults were 1.48 (95% CI: 1.02~2.14) and 1.83 (95% CI: 1.23~2.72). **Figure 2** illustrates a forest plot of values after adjusting for confounding factors for sex and age.

**Table 6:** CVD incidence according to the baseline BRI in CircS patients, stratified by sex and age.

	OR (95% CI)	*P value*	*P* for interaction
**Sex**			0.031
Male			
Q1 (< 4.00)	Reference		
Q2 (4.00 ~ 4.82)	1.39 (0.91~2.10)	0.124	
Q3 (4.82 ~ 5.65)	1.72 (1.12~2.64)	0.014	
Q4 (> 5.65)	2.59 (1.55~4.33)	<0.001	
Female			
Q1 (< 4.00)	Reference		
Q2 (4.00 ~ 4.82)	1.22 (0.86~1.73)	0.271	
Q3 (4.82 ~ 5.65)	1.10 (0.78~1.55)	0.595	
Q4 (> 5.65)	1.32 (0.95~1.84)	0.094	
**Age**			0.811
<60 years			
Q1 (< 4.00)	Reference		
Q2 (4.00 ~ 4.82)	1.09 (0.76~1.56)	0.658	
Q3 (4.82 ~ 5.65)	1.37 (0.96~1.96)	0.083	
Q4 (> 5.65)	1.48 (1.02~2.14)	0.040	
>=60 years			
Q1 (< 4.00)	Reference		
Q2 (4.00 ~ 4.82)	1.70 (1.13~2.55)	0.010	
Q3 (4.82 ~ 5.65)	1.35 (0.90~2.03)	0.146	
Q4 (> 5.65)	1.83 (1.23~2.72)	0.003	

CVD, cardiovascular disease; CircS, circadian syndrome; BRI, Body-roundness index; OR, odds ratio; CI, confidence interval.

Adjusted for age, sex, education level, residence, marital status, smoking status, alcohol consumption, physical activity.

### 3.6 Sensitivity analysis

Furthermore, a sensitivity analysis showed results similar to the primary analysis. When excluding those participants with less than 2 years of follow-up, we observed results similar to the association between BRI and CVD incidence in CircS patients, as shown in **Supplementary Table 2**. The associations between incident CVD and the fourth BRI quartiles were 1.59 (95% CI: 1.21~2.1), relative to the first BRI quartile.

## 4 Discussion

In this study, we investigated the relationship between CircS, BRI, and CVD among middle-aged and elderly individuals. We found that BRI was independently associated with CircS, outperforming obesity indices such as BMI and WC in identifying individuals with CircS. During the 9-year follow-up, we discovered a significant association between elevated BRI and CVD incidence, especially among men with CircS.

This is the first study to reveal an independent association between BRI and CircS. In this study, the prevalence of CircS was 40.3%, consistent with previous research findings (5). CircS, based on metabolic syndrome and associated with circadian rhythm disturbances, represented a new CVD risk cluster (4). The other two components—reduced sleep duration and depression—were also linked to CVD (23, 24). Additionally, as a new anthropometric index, BRI provided a better prediction of body fat and visceral adipose tissue volume (11). Some research has indicated that BRI could serve as a fat indicator to determine the presence of hyperuricemia (25, 26), arterial stiffness (27–29), CVD (30, 31), diabetes (25, 26, 32, 33), dyslipidemia (34, 35), and hypertension (36, 37). An extensive meta-analysis revealed that BRI demonstrated strong predictive ability for metabolic syndrome across various nationalities and races, outperforming traditional indices like BMI, WHR, ABSI, and BAI in predicting metabolic syndrome (38). Recent studies have also shown an association between BRI levels and depressive symptoms (39, 40). Additionally, a meta-analysis of prospective cohort studies revealed that compared to normal sleep duration, short sleep duration (defined in most studies as less than 5 or 6 hours per day) was linked to a 38% absolute increase in the incidence of obesity (41). Further evidence has suggested that insufficient sleep and circadian rhythm disruption contributes to poor metabolic health and obesity by altering various components of energy metabolism and behavior (42). In addition, obese individuals frequently report issues with sleep duration and quality (43), which may be linked to behavioral factors such as poor diet and lack of physical exercise among this population (42). Our study revealed that BRI, when compared to obesity indices such as BMI and WC, exhibits a higher AUC and a stronger overall discriminative capacity, showing exceptional performance in CircS diagnosis among middle-aged and elderly Chinese individuals. This indicates that BRI, as a quantitative indicator of visceral fat, is a more reliable marker for CircS than general obesity indices.

Our research revealed a significant correlation between elevated BRI levels and CVD incidence in CircS patients. Compared to the first quartile, individuals in the third and fourth quartiles of BRI have a higher risk of CVD occurrence. This suggests that even when considering the circadian rhythm’s predictive role in CVD occurrence, the effect of abdominal obesity should not be ignored. Previous studies have indicated that visceral adipose tissue is an important predictor of cardiovascular risk. BRI has been shown to improve the ability to predict body fat and visceral adipose tissue, thereby better reflecting the body’s health status (11). It has also been found that BRI has a U-shaped relationship with all-cause mortality and cardiovascular mortality (15). This could be due to the significant association between BRI and insulin resistance (38), which leads to imbalances in glucose and lipid metabolism, oxidative stress, inflammation, and vascular endothelial cell damage (44). Additionally, we discovered that among CircS patients, other obesity indices, such as BMI, are significantly associated with CVD occurrence only in the fourth quartile, with no statistical significance observed in the second or third quartiles. This could be due to the closer relationship between abdominal obesity and disruptions in circadian rhythms (45, 46), as well as cardiovascular disease, compared to general obesity. Moreover, circadian rhythm disturbances could disrupt lipid and glucose metabolism, gut microbiota, and the neuroendocrine regulation of appetite. Sleep deprivation exacerbated by circadian rhythm disruptions may also lead to unhealthy eating habits, resulting in metabolic disorders and ultimately obesity, especially abdominal obesity (42).

Of note, we found that men with CircS who had elevated BRI levels have a higher risk of developing CVD. In this study, the proportion of females in the CircS group was higher, and among CircS patients, the proportion of females increased with higher BRI levels. However, existing evidence on differences in circadian rhythm disturbances and obesity between the sexes is limited and sometimes conflicting (47), calling for more rigorous evidence exploring their sex-specificity. It has been established that men with CircS have a higher risk of CVD than women (5, 21). In this study, we considered that this may be related to the cardioprotective effect of estrogen under circadian disruption. It has been found that circadian disruption has a significant sex-dependent effect on glucose and energy metabolism (48), leading to abnormal levels of the satiety hormone leptin, the respiratory quotient and the hunger hormone ghrelin (49), with disturbances in these processes being risk factors for obesity and CVD. Male gonadal hormones exacerbate these metabolic effects, while female gonadal hormones can mitigate them (50). However, further evidence needed to investigate the potential mechanisms of sex hormones in abdominal obesity and CVD under circadian disruption remains lacking.

Our study has several advantages in terms of design and strategy. Firstly, we employed a comprehensive dataset from a nationally representative epidemiological survey, characterized by a large sample size and a prolonged follow-up period. This robust dataset allowed us to generalize our findings to the broader middle-aged and elderly population in China. Additionally, this is the first study to investigate the association between BRI and CVD occurrence in CircS patients, and the results were stratified to explore the effects of sex and age on the outcomes. The results indicated that CircS and BRI should be incorporated into the early warning and prevention of cardiovascular disease in middle-aged and elderly individuals.

Our observational study also has several limitations. Firstly, even though we adjusted for many covariates, unmeasured factors may still have biased the results. Additionally, CVD diagnosis relied on self-reported physician diagnoses rather than medical records, which may have introduced some deviation. However, self-reported CVD has been shown to be reliable in ascertaining non-fatal events (51, 52). Additionally, other large-scale studies have demonstrated notable agreement between self-reported CVD and medical records (53). Therefore, we interpreted our findings with caution and recommend further prospective studies of larger populations to improve the accuracy and reliability of the results.

## 5 Conclusions

In our study, BRI was independently associated with CircS, outperforming obesity indices such as BMI and WC in identifying individuals with CircS. During the 9-year follow-up, elevated BRI levels was significantly associated with CVD incidence in CircS patients, especially among men. These observations have important implications for current CVD management strategies, indicating that BRI can be an excellent tool for screening CircS. Thus, early identification of high-risk populations with CircS and elevated BRI levels may help promote healthy aging in middle-aged and elderly individuals.

## Data Availability

The datasets presented in this study can be found in online repositories. The names of the repository/repositories and accession number(s) can be found below: China Health and Retirement Longitudinal Study (CHARLS)(http://charls.pku.edu.cn/).
