# Supplementary material for: Elevated body roundness index increases the risk of cardiovascular disease in Chinese patients with circadian syndrome
Source: Front Endocrinol (Lausanne). 2025 Feb 25;16:1532344. doi: 10.3389/fendo.2025.1532344 (PMC11893425; doi:10.3389/fendo.2025.1532344)
Supplement: Supplementary file 1 [file DataSheet1.docx]

Supplementary Material

# Supplementary Table

Supplementary Table 1. CVD incidence according to CircS patients’ baseline adiposity indices, 2011–2020

| **Outcome** | **Case,%** |  |  | **OR (95%CI)** |  |  |  |
| --- | --- | --- | --- | --- | --- | --- | --- |
|  |  | **Model 1** | ***P* value** | **Model 2** | ***P* value** | **Model 3** | ***P* value** |
| **CVAI** |  |  |  |  |  |  |  |
| Per SD increase | 680 (29.3) | 1.17 (1.07~1.29) | 0.001 | 1.15 (1.05~1.26) | 0.004 | 1.14 (1.03~1.25) | 0.009 |
| Quartiles |  |  |  |  |  |  |  |
| Q1 | 149 (25.8) | Reference | | Reference | | Reference | |
| Q2 | 161 (27.9) | 1.11 (0.86~1.44) | 0.426 | 1.09 (0.84~1.42) | 0.499 | 1.08 (0.83~1.41) | 0.563 |
| Q3 | 177 (30.6) | 1.27 (0.98~1.64) | 0.067 | 1.23 (0.95~1.59) | 0.115 | 1.19 (0.92~1.54) | 0.192 |
| Q4 | 193 (33.4) | 1.44 (1.12~1.86) | 0.005 | 1.38 (1.07~1.79) | 0.014 | 1.34 (1.03~1.73) | 0.029 |
| *P* for trend |  |  | 0.003 |  | 0.009 |  | 0.021 |
|  |  |  |  |  |  |  |  |
| **BMI** |  |  |  |  |  |  |  |
| Per SD increase | 520 (22.4) | 1.12 (1.01~1.24) | 0.026 | 1.11 (1~1.23) | 0.053 | 1.09 (0.98~1.21) | 0.104 |
| Quartiles |  |  |  |  |  |  |  |
| Q1 | 155 (26.8) | Reference | | Reference | | Reference | |
| Q2 | 157 (27.2) | 1.02 (0.78~1.32) | 0.895 | 0.93 (0.7~1.24) | 0.635 | 1.08 (0.83~1.41) | 0.577 |
| Q3 | 170 (29.4) | 1.14 (0.88~1.47) | 0.327 | 1.07 (0.81~1.42) | 0.632 | 1.25 (0.96~1.63) | 0.104 |
| Q4 | 198 (34.3) | 1.42 (1.11~1.83) | 0.006 | 1.24 (0.94~1.63) | 0.132 | 1.54 (1.18~2) | 0.001 |
| *P* for trend |  |  | 0.004 |  | 0.082 |  | 0.01 |
|  |  |  |  |  |  |  |  |
| **WC** |  |  |  |  |  |  |  |
| Per SD increase | 520 (22.4) | 1.12 (1.01~1.24) | 0.026 | 1.11 (1~1.23) | 0.053 | 1.09 (0.98~1.21) | 0.104 |
| Quartiles |  |  |  |  |  |  |  |
| Q1 | 148 (25.5) | Reference | | Reference | | Reference | |
| Q2 | 158 (27.2) | 1.09 (0.84~1.42) | 0.505 | 0.93 (0.7~1.24) | 0.635 | 1.11 (0.85~1.45) | 0.431 |
| Q3 | 151 (27.2) | 1.09 (0.84~1.42) | 0.518 | 1.07 (0.81~1.42) | 0.632 | 1.11 (0.85~1.44) | 0.461 |
| Q4 | 223 (36.7) | 1.7 (1.32~2.17) | <0.001 | 1.24 (0.94~1.63) | 0.132 | 1.72 (1.33~2.22) | <0.001 |
| *P* for trend |  |  | <0.001 |  | 0.082 |  | <0.001 |

CVD: cardiovascular disease; CircS: circadian syndrome; CVAI: Chinese visceral adiposity index; BMI: body mass index; WC: waist circumference; OR: odds ratio; CI: confidence interval.

Model 1: unadjusted. Model 2: adjusted for age, sex. Model 3: adjusted for age, sex, educational level, residence, marital status, smoking status, alcohol consumption, physical activity.

Supplementary Table 2. CVD incidence according to baseline BRI in CircS patients in sensitivity analyses.

| **Outcome** | **Case,%** |  |  | **OR (95%CI)** |  |  |  |
| --- | --- | --- | --- | --- | --- | --- | --- |
|  |  | **Model 1** | ***P* value** | **Model 2** | ***P* value** | **Model 3** | ***P* value** |
| **CVD** |  |  |  |  |  |  |  |
| Per SD increase | 647 (29.2) | 1.20 (1.09~1.31) | <0.001 | 1.17 (1.06~1.29) | 0.001 | 1.15 (1.05~1.27) | 0.004 |
| Quartiles |  |  |  |  |  |  |  |
| Q1 | 131 (23.7) | Reference | | Reference | | Reference | |
| Q2 | 157 (28.4) | 1.28 (0.98~1.68) | 0.072 | 1.30 (0.99~1.70) | 0.061 | 1.26 (0.96~1.66) | 0.094 |
| Q3 | 166 (30) | 1.38 (1.05~1.80) | 0.019 | 1.36 (1.04~1.79) | 0.024 | 1.34 (1.02~1.76) | 0.035 |
| Q4 | 193 (34.9) | 1.73 (1.33~2.25) | <0.001 | 1.67 (1.27~2.19) | <0.001 | 1.59 (1.21~2.10) | 0.001 |
| *p* for trend |  |  | <0.001 |  | <0.001 |  | 0.001 |
|  |  |  |  |  |  |  |  |
| **Heart disease** |  |  |  |  |  |  |  |
| Per SD increase | 492 (22.2) | 1.17 (1.05~1.29) | 0.003 | 1.11 (1~1.24) | 0.043 | 1.10 (0.99~1.22) | 0.079 |
| Quartiles |  |  |  |  |  |  |  |
| Q1 | 102 (18.4) | Reference | | Reference | | Reference | |
| Q2 | 118 (21.4) | 1.20 (0.89~1.62) | 0.223 | 1.18 (0.88~1.59) | 0.279 | 1.15 (0.86~1.56) | 0.347 |
| Q3 | 123 (22.2) | 1.26 (0.94~1.69) | 0.121 | 1.19 (0.89~1.61) | 0.246 | 1.17 (0.87~1.58) | 0.294 |
| Q4 | 149 (26.9) | 1.63 (1.23~2.17) | 0.001 | 1.46 (1.09~1.96) | 0.012 | 1.41 (1.04~1.89) | 0.025 |
| *p* for trend |  |  | 0.001 |  | 0.016 |  | 0.031 |
|  |  |  |  |  |  |  |  |
| **Stroke** |  |  |  |  |  |  |  |
| Per SD increase | 233 (10.5) | 1.13 (0.99~1.30) | 0.077 | 1.17 (1.01~1.35) | 0.034 | 1.16 (1.00~1.34) | 0.045 |
| Quartiles |  |  |  |  |  |  |  |
| Q1 | 47 (8.5) | Reference | | Reference | | Reference | |
| Q2 | 57 (10.3) | 1.24 (0.83~1.86) | 0.299 | 1.33 (0.88~2.00) | 0.175 | 1.29 (0.85~1.95) | 0.228 |
| Q3 | 62 (11.2) | 1.36 (0.91~2.02) | 0.134 | 1.47 (0.98~2.21) | 0.061 | 1.46 (0.97~2.19) | 0.069 |
| Q4 | 67 (12.1) | 1.48 (1.00~2.20) | 0.049 | 1.67 (1.11~2.53) | 0.014 | 1.62 (1.07~2.46) | 0.023 |
| *p* for trend |  |  | 0.045 |  | 0.013 |  | 0.019 |

CVD, cardiovascular disease; CircS, circadian syndrome; BRI, body-roundness index; OR, odds ratio; CI, confidence interval.

Model 1: unadjusted. Model 2: adjusted for age, sex. Model 3: adjusted for age, sex, educational level, residence, marital status, smoking status, alcohol consumption, physical activity.

# Supplementary Figures


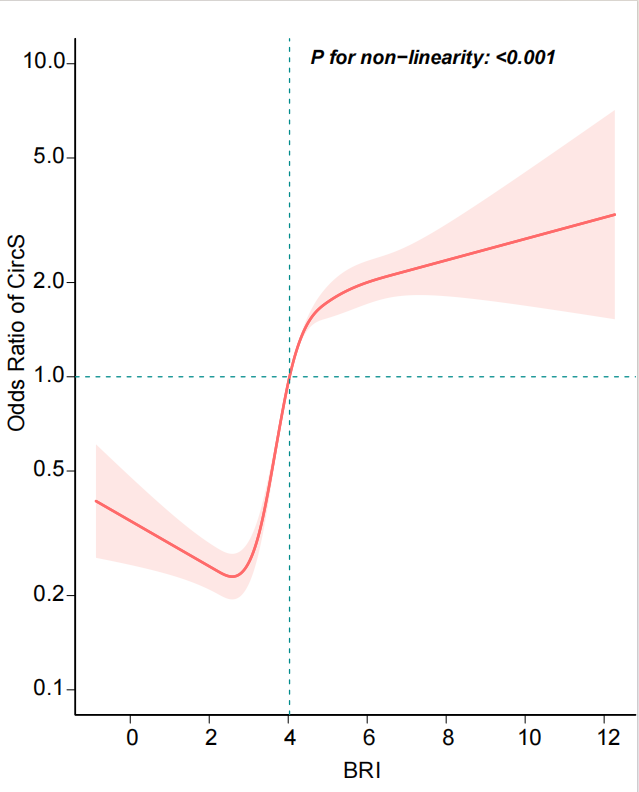


Supplementary Figure 1. Dose–response relationship of baseline BRI with CircS. BRI: body-roundness index; CircS: circadian syndrome

**
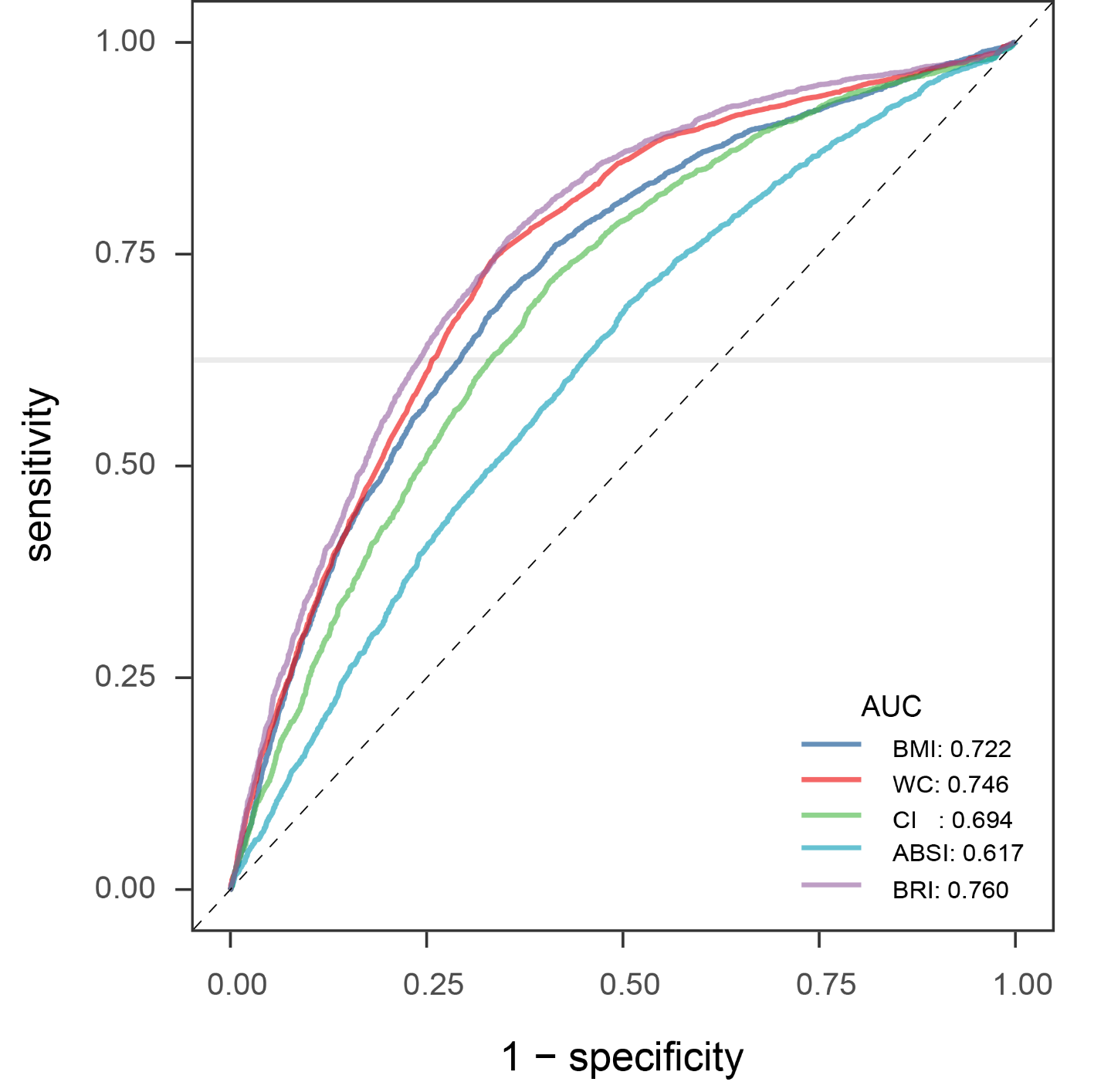
**

Supplementary Figure 2. ROC curves for the adiposity indices for CircS diagnosis


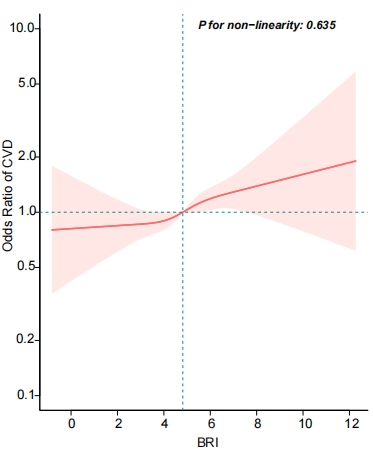


Supplementary Figure 3. Dose–response relationship of BRI and CVD incidence in CircS patients. CVD: cardiovascular disease; CircS: circadian syndrome; BRI: body-roundness index


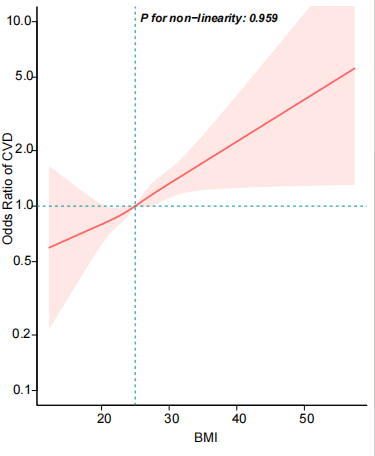


Supplementary Figure 4. Dose–response relationship of BMI and CVD incidence in CircS patients. CVD: cardiovascular disease; CircS: circadian syndrome; BMI: body mass index


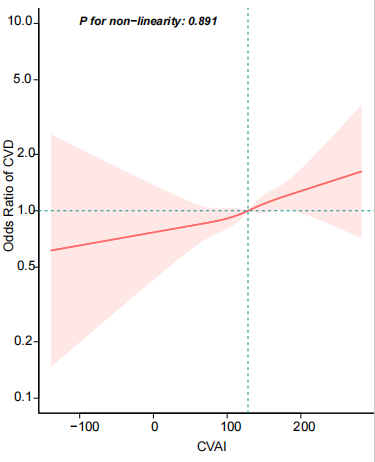


Supplementary Figure 5. Dose–response relationship of CAVI and CVD incidence in CircS patients. CVD: cardiovascular disease; CircS: circadian syndrome; CVAI: Chinese visceral adiposity index


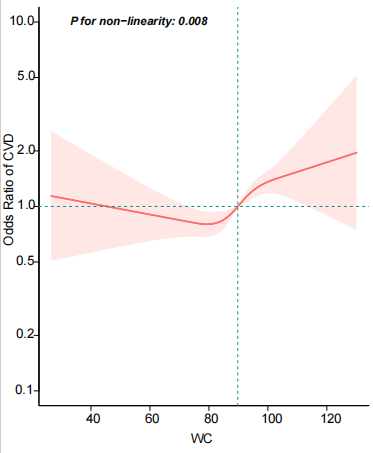


Supplementary Figure 6. Dose–response relationship of WC and CVD incidence in CircS patients. CVD: cardiovascular disease; CircS: circadian syndrome; WC: waist circumference
